# Investigation of Metabolome Underlying the Biological Mechanisms of Acute Heat Stressed Granulosa Cells

**DOI:** 10.3390/ijms23042146

**Published:** 2022-02-15

**Authors:** Abdul Sammad, Lirong Hu, Hanpeng Luo, Zaheer Abbas, Saqib Umer, Shanjiang Zhao, Qing Xu, Adnan Khan, Yajing Wang, Huabin Zhu, Yachun Wang

**Affiliations:** 1 National Engineering Laboratory for Animal Breeding, Key Laboratory of Animal Genetics, Breeding and Reproduction, MARA, College of Animal Sciences and Technology, China Agricultural University, Beijing 100193, China; drabdulsammad1742@yahoo.com (A.S.); 15121578@bjtu.edu.cn (L.H.); luohanpeng@cau.edu.cn (H.L.); zaheerabbas@bjtu.edu.cn (Z.A.); dr.adnan93@cau.edu.cn (A.K.); yajingwang@cau.edu.cn (Y.W.); 2 Embryo Biotechnology and Reproduction Laboratory, Institute of Animal Sciences, Chinese Academy of Agricultural Sciences, Beijing 100193, China; saqibumar33@gmail.com (S.U.); zhaoshanjiang@caas.cn (S.Z.); 3 College of Life Sciences and Bioengineering, Beijing Jiaotong University, Beijing 100044, China; qingxu@bjtu.edu.cn

**Keywords:** granulosa cells, heat stress, metabolomics, metabolites, bioenergetics, pathways

## Abstract

Heat stress affects granulosa cells and the ovarian follicular microenvironment, ultimately resulting in poor oocyte developmental competence. This study aims to investigate the metabo-lomics response of bovine granulosa cells (bGCs) to in vitro acute heat stress of 43 °C. Heat stress triggers oxidative stress-mediated apoptosis in cultured bGCs. Heat-stressed bGCs exhibited a time-dependent recovery of proliferation potential by 48 h. A total of 119 metabolites were identified through LC–MS/MS-based metabolomics of the spent culture media, out of which, 37 metabolites were determined as differentially involved in metabolic pathways related to bioenergetics support mechanisms and the physical adaptations of bGCs. Multiple analyses of metabolome data identified choline, citric acid, 3-hydroxy-3-methylglutaric acid, glutamine, and glycocyamine as being upregulated, while galactosamine, AICAR, ciliatine, 16-hydroxyhexadecanoic acid, lysine, succinic acid, uridine, xanthine, and uraconic acid were the important downregulated metabolites in acute heat stress. These differential metabolites were implicated in various important metabolic pathways directed towards bioenergetics support mechanisms including glycerophospholipid metabolism, the citrate cycle (TCA cycle), glyoxylate and dicarboxylate metabolism, and serine, threonine, and tyrosine metabolism. Our study presents important metabolites and metabolic pathways involved in the adaptation of bGCs to acute heat stress in vitro.

## 1. Introduction

Heat stress deserves more attention as a climate change impact of unregulated global warming [1] and presents an important challenge to food security [2], public health [3], and sustainable dairy farming [4,5]. Usually, at an ambient temperature exceeding 25 °C, all living organisms—including cattle—experience heat stress [6,7,8], while higher temperatures in the range of 35–40 °C cause significant physiological and biochemical changes in the body [7,9,10]. Heat stress causing biochemical alterations in the maternal reproductive system has been attributed to low conception rates and higher embryonic losses in cattle [9,10,11].

The granulosa cells present in ovarian follicles are important for oocyte development due to their specialized steroidogenesis role and cross talk with oocytes [12,13]. Heat stress significantly decreases bovine granulosa cell (bGCs) survival, inhibits steroidogenesis, limits proliferation and cell transition, increases oxidative stress, and promotes apoptosis in bGCs [14,15,16]. The impairment of granulosa cells through environmental or physiological stress leads to the disruption of ovarian activity and oocyte development competence [17,18]. In a cohort study, high ambient temperatures were associated with diminished ovarian reserves and reproductive aging in women [19]. Increased rates of granulosa cells apoptosis are particularly associated with diminished ovarian reserves and poor fertility outcomes [20]. The pre-ovulatory stages of ovarian follicles are more susceptible to heat stress, leading to oxidative stress and damage to the contained oocytes [21,22,23]. Heat stress is also shown to speed up the process of leutinization of granulosa cells, which has been associated with reduced fertility [24]. Heat stress triggers a series of energetic metabolism changes in the body [25], high non-esterified fatty acids, ketone bodies, induction of inflammatory cytokines, and dynamic role of different metabolites can alter the ovarian follicular biochemical profile [10,23,26,27,28,29]. This is why heat stress-mediated alterations of the follicular biochemical profile have been associated with granulosa cell dysfunction and poor oocyte developmental competence [25,30,31,32,33]. Interestingly, in our previous study [15], 41 °C appeared to be slightly less lethal than 40 °C to bGCs. Additionally, there have been studies investigating heat stress on granulosa cells within the range of 41 °C [14], 42 °C [30], and 43 °C [31]. All of these studies used the duration of 2 h for heat stress, and the results were more or less consistent among these studies on the physical parameters of heat-stressed granulosa cells. Hence, this study selected 43 °C for a duration of 2 h to confirm the consistency of the physical insult to the granulosa cells (with the mentioned previous studies) and collect samples for further investigation.

Transcriptome level studies have given essential insights into the mechanisms of granulosa cell apoptosis; however, there is also evidence of improved heat shock protein responses and upregulation of certain components of metabolic and antioxidant pathways [15]. A recent study of acute heat-stressed granulosa cells suggested several proteins and peptide signatures involved in extensive remodeling of the cell cytoskeleton [32]. There is evidence of the proliferation senescence of granulosa cells after heat stress and their ability to recover their proliferation potential following both acute and chronic heat stress [32,33]. Metabolomics investigations are a proven, mature technique, helping researchers to unlock potential causative metabolites and metabolic pathways [34,35]. In fact, a recent study suggested that even metabolites in themselves can respond to heat stress by employing protective responses to cellular stress [29]. To the best of our knowledge, there is a lack of systematic metabolomics studies of granulosa cell cultures in response to acute heat stress. We hypothesize that bGCs exposed to acute heat stress will experience physical insults and employ key metabolites and metabolic pathways involved in cellular homeostatic mechanisms. This study evaluates the physiological parameters of the granulosa cell and cell culture metabolomics landscape in response to in vitro acute heat stress.

## 2. Results

The control group refers to bGCs maintained at 38 °C, while the treatment or heat stress group mentions the bGCs exposed to 43 °C for 2 h. In this section, the word “cells” refers to bGCs.

### 2.1. Influence of Heat Stress on the Physical Parameters of Bovine Granulosa Cells

No change in cell viability was observed until 24 h after heat stress exposure (Supplementary Figure S1A). Cells in the control group maintained steady proliferation activity (Supplementary Figure S1A). A slight positive recovery was observed after 24 h post-exposure in the treatment group, while cells in both groups proliferated significantly after the culture medium change at 48 h post-exposure (Supplementary Figure S1A). Cell confluence was attained at 72 h post-exposure in both the control and treatment groups. The medium change at 96 h had no further effect on cell proliferation. Similarly, cells were allowed a post-heat stress exposure recovery period of 6 h at 38 °C [30,36], followed by the measurement of cellular ROS (reactive oxygen species) and apoptosis. A significantly (*p*-value < 0.05) increased level of intracellular ROS was observed in the heat stress group compared to the control group (Supplementary Figure S1B). Heat-stressed cells exhibited increased apoptosis compared to the control group, as shown in representative florescent microphotographs in Supplementary Figure S1C,D, respectively. The apoptotic rate (early apoptotic + late apoptotic) of the cells was significantly (*p*-value < 0.05) higher in the treatment group (Supplementary Figure S1E).

### 2.2. Metabolome Profile in the Culture Medium of Bovine Granulosa Cells

**Figure 1:**
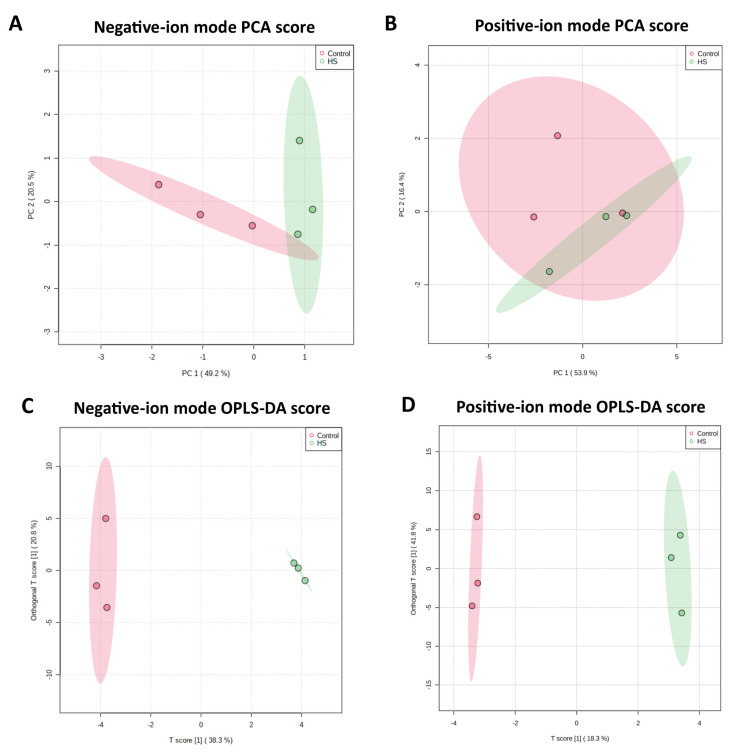
Results of principal component analysis (PCA) are presented for the metabolite sets in three replicates each of the control (red) and heat stress (green) groups in negative ion mode (**A**) and positive ion mode (**B**) of LC–MS/MS analysis. Likewise, the results of orthogonal partial least squares discrimination analysis (OPLS-DA) score for both ion modes’ (**C**,**D**) metabolites are given.

Metabolome data was obtained in triplicate for the spent culture medium of the cells from the control and heat stress groups through LC–MS/MS. The total ion current diagrams of all the quality control samples from both the positive ion (POS) and negative ion (NEG) modes were superimposed, where the spectrum overlapped well and the retention time and peak signal intensity fluctuations were small for both ion modes (Supplementary Figure S2A,B), showing a good stability and repeatability accuracy of the employed LC–MS protocol. What is more, the MS2 component (2056 and 2597 MS2 spectra in POS and NEG modes, respectively) from the samples was plotted against the distribution of the mass-to-charge ratio and retention time (Supplementary Figure S2C,D), which indicated that the peaks’ capacity was good and the obtained data was reliable. Based on the metabolites’ identification in the MSBank and KEGG databases, 71 and 48 metabolites were finally determined in POS and NEG mode, respectively. Metabolite tables of both the NEG and POS modes with samples in rows and metabolites in columns were separately subjected to principal component analysis (PCA) analysis (Figure 1A,B), which displays a clear separation between the two groups (control and heat stress groups).

### 2.3. Differential Metabolites between Control and Heat Stress Groups

As shown in the orthogonal partial least squares-discriminate analysis (OPLS-DA) score presented in Figure 1C,D, the optimum distinction between the control and heat stress groups was achieved in both ion modes. The orthogonal T-score for the first component stood at around 60% for both NEG and POS mode metabolites, showing good variance between the two groups, supporting a recommendation of OPLS-DA for variable metabolomics data sets [37]. The differential metabolites between the two groups were determined through the criterion of an OPLS-DA’s VIP (variable importance in the projection) score > 1. Around 37 differential metabolites were identified as being differential, as presented in Table 1, where the majority of them belong to amino acid, carbohydrate, and fatty acid metabolism.

**Figure 2:**
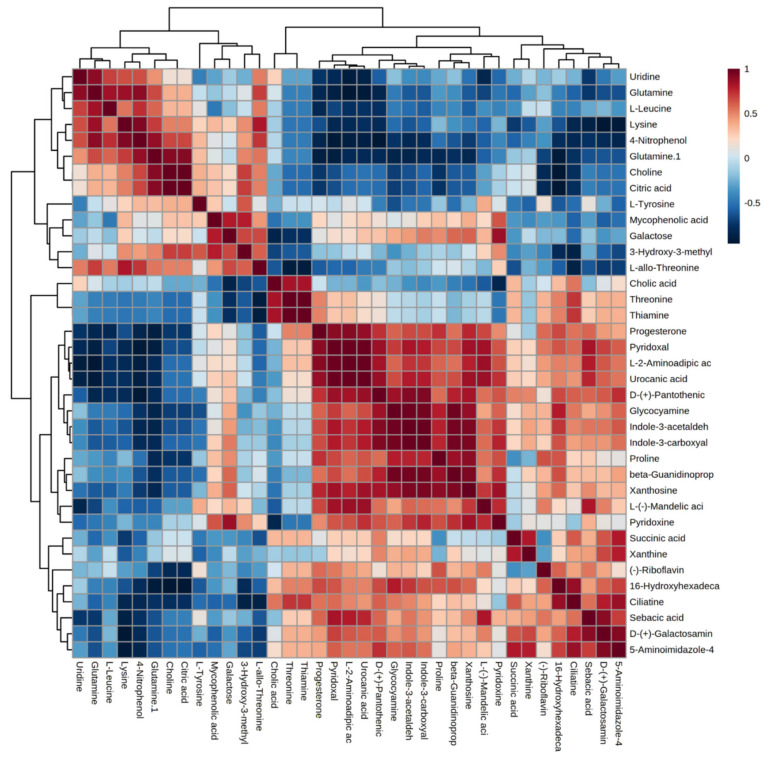
Pearson’s correlation heatmap along with clustering patterns among the 37 differential metabolites with a variable importance in the projection (VIP) score of more than 1.

A correlation heatmap of the differential metabolites is given in Figure 2, where the positive and negative correlations are distinctly shown based on color variation, while the clustering patterns among the metabolite sets are also visualized. The correlation map shows the extensive metabolism of amino acids and the enrichment of metabolite oxidation for the energy support of the heat-stressed bGCs.

**Figure 3:**
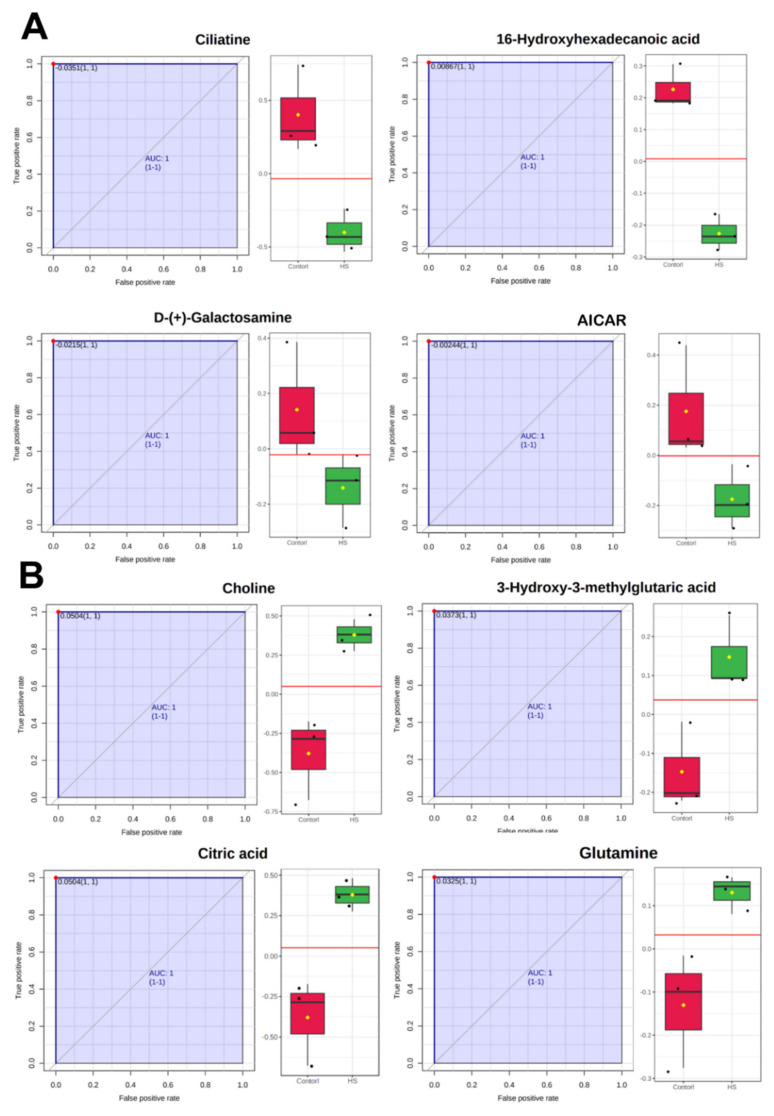
Biomarkers and performance evaluation of metabolites obtained through a receiver operator characteristic (ROC) analysis along with the regulation status of each metabolite. ROC curves with area under curve (AUC) are plotted with sensitivity values on the y-axis and the corresponding false positive rate on the x-axis. Upper panel (**A**) represents significant upregulated metabolites while the lower panel (**B**) shows the significant downregulated metabolites.

Differential metabolites were subjected to ROC (receiver operator characteristic) analysis to determine their biomarker prediction values (results of AUC, *p*-values, and FC presented in Supplementary Table S1). Paired sensitivity and false-positive ratios at different classification decision boundaries were calculated. A ROC curve was plotted with a sensitivity value on the y-axis and the corresponding false positive rate on the x-axis. Metabolites with an AUC = 1 (area under curve equal to 1 presents excellent biomarker prediction) included choline, 3-hydroxy-3-methylglutaric acid, 16-hydroxyhexadecanoic acid, galactosamine, ciliatine, AICAR (5-Aminoimidazole-4-carboxamide-1-beta-D-ribofuranosyl 5’-monophosphate), and citric acid—where choline, 3-hydroxy-3-methylglutaric acid, citric acid were highly upregulated in the heat stress group (Figure 3B), and 16-Hydroxyhexadecanoic acid, galactosamine, ciliatine, and AICAR were downregulated in the heat stress group (Figure 3A).

Furthermore, differential metabolites were subjected to DSPC (Debiased Sparse Partial Correlation) correlation network analysis (Table 2). Important correlation nodes from the network analysis included glutamine, 4-nitrophenol, uraconic acid, L-2-aminoadipic acid, uridine, L-leucine, lysine, xanthosine, succinic acid, progesterone, xanthine, and citric acid. The results of the network analysis augmented the results of the differential metabolites based on their VIP values, whereas it helped to segregate important central metabolites among the list of differential metabolites defining the core of metabolome changes involved in heat-stressed bGCs.

### 2.4. Metabolic Pathways Involved in the Heat Stress of Bovine Granulosa Cells

**Figure 4:**
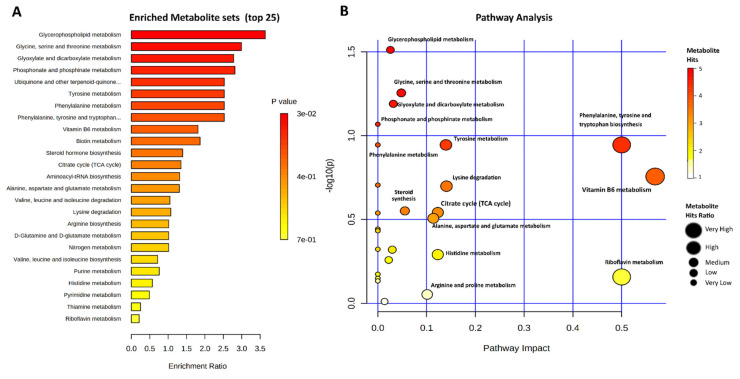
Enrichment analysis (**A**) and pathway analysis (**B**) showing the biological processes of differential metabolite involvement in heat-stressed bGCs. In the enrichment analysis (**A**), the length of the bar along the x-axis shows the enrichment ratio among particular metabolite sets, while the color bar represents the *p*-value. The pathway analysis (**B**) shows the pathways’ impact on the x-axis and the *p*-value on the y-axis, while the color bar legend shows the raw *p*-value distribution and the dot size shows the metabolite hit ratios in the given pathways.

The top 25 enriched metabolite sets and their related pathways are presented in Figure 4A,B, respectively, while details are presented in Supplementary Table S2. The Glycerophospholipid metabolism and Glycine, serine, and threonine metabolism sets’ enrichment ratio was ≥3. The following six metabolites sets had an enrichment ratio of ≥2.5, while for Vitamin B6 metabolism and Biotin metabolism the enrichment ratio was ≥2. The remaining metabolite sets had an enrichment ratio <2, as shown in Figure 4A. Three metabolites sets and their corresponding metabolic pathways—namely, “Glycine, serine and threonine metabolism”, “Glycerophospholipid metabolism”, and “Glyoxylate and dicarboxylate metabolism”—were significantly enriched among the differential metabolites. In the case of Glycine, serine, and threonine metabolism, four differential metabolites—choline, glycocyamine, threonine, and L-allo-threonine—were involved. Choline was additionally enriched in Glycerophospholipid metabolism. Succinic acid (enriched in Propanoate metabolism) and citric acid (enriched in Glyoxylate and dicarboxylate metabolism) were jointly involved in the Citrate (TCA) cycle and Alanine, aspartate and glutamate metabolism enrichment sets and pathways. Ciliatine was enriched in the Phosphonate and phosphinate metabolism pathway. L-2-Aminoadipic acid and Lysine were enriched in the Lysine degradation pathway. Xanthine, AICAR, and xanthosine were involved in the Purine metabolism pathway. Along with glutamine, uridine and urocanic acid were enriched in the Pyrimidine and Histidine metabolism pathways, respectively. Glutamine was also additionally involved in three other metabolic pathways. Indole-3-acetaldehyde, serotonin, and L-tyrosine were enriched in the Phenylalanine, tyrosine and tryptophan biosynthesis pathway. Progesterone was involved in Steroid hormone biosynthesis. Additionally, six differential metabolites (all amino acids) were found to be enriched in the Aminoacyl-tRNA biosynthesis metabolic pathway.

## 3. Discussion

Heat stress has been associated with a variety of physiological and metabolic modifications in cows [38,39]. The early postpartum achievement of conception is imperative for profitable dairy farming. However, postpartum cows can suffer from a negative energy balance, which causes lipolysis of body fat reserves in the form of non-esterified fatty acids [40,41,42]. This phenomenon, when accompanied with low body condition scores, clinical ketosis, decreased feed intake, fatty liver, and other morbidities, leads to impairment of the reproduction process [43,44,45]. Additionally, summertime heat stress in the presence of these conditions can further exaggerate this metabolic stress, leading to low reproductive performance [46,47]. Therefore, it was imperative to investigate the physical and biochemical adaptation, in conjunction with the metabolome changes, in in vitro acute heat-stressed bGCs. Cell proliferation activity significantly declined in response to acute heat stress treatment (43 °C). while the control group (38 °C) of bGCs steadily proliferated. This finding is in accordance with previous studies, where acute heat-stressed (45 °C) granulosa cells resumed proliferation after the 48 h recovery period [31,32,33]. Moreover, acute heat stress triggered intracellular ROS accumulation and apoptosis in bGCs; these results are in accordance with prior studies on heat stress in bGCs [15,30]. Higher ROS production can impair cellular antioxidant defense systems, leading to oxidative stress [48]. Heat stress-associated cellular ROS triggers oxidative impairment [49] and apoptosis of cells [50,51]. ROS-mediated oxidative stress can induce bGC apoptosis and compromised fertility or fertility failure in cows [52,53]. However, the mechanisms and pathways through which heat stress causes extreme oxidative stress and decreases in antioxidant defense responses leading to apoptosis are not completely understood. We propose further transcriptome investigations investigating the underlying bGC bioenergetics-based protective responses against thermally driven oxidative stress and recovery mechanisms.

While much has been known about the physical and transcriptional changes of bGCs in response to heat stress [15,32], our LC–MS/MS-based untargeted metabolomics study has revealed important insights into the biological mechanisms of bGC adaptation to acute heat stress. Thirty-seven differential metabolites were identified, and to the best of our knowledge, this is the first metabolic profiling of the culture media of heat-stressed bGCs—a helpful simulation of the follicular microenvironment. Some of the most abundant molecules were involved in amino acid, carbohydrates, and lipid metabolism. Furthermore, this metabolome data also provides basic information on metabolite enrichment in bGCs under thermoneutral culturing conditions. A total of 119 unique metabolites were detected through LC–MS/MS—out of which, 37 metabolites were considered to be differentially expressed. Following this, we discuss a brief overview of these metabolites and their regulation. Mycophenolic acid (upregulated in heat stress), belonging to the inositol phosphate pathway, is a potent inhibitor of purine synthesis and is primarily involved in immunosuppression [54]. Following a previous study, choline (Phospholipids and Phospholipid Metabolism) was also upregulated in heat stress [55]. Additionally, 3-hydroxy-3-methylglutaric acid—a dicarboxylic acid implicated in the leucine pathway—was upregulated in heat-stressed bGCs. This metabolite contributes positively to the bioenergetic metabolism of cells in the presence of mitochondrial function disruption under heat stress [56,57]. Most importantly, citric acid (central to the tricarboxylic acid (TCA) cycle) was upregulated in heat-stressed bGCs, which are involved in the catabolism of fatty acids, carbohydrates, and amino acids, presenting a classical example of catabolic activity in heat-stressed cells. Citric acid is central to diverse cellular energy support [58], helpful in relieving oxidative stress [59], and in the relief of heat stress in tissues [60]. Meanwhile, succinic acid—as the intermediate of citric acid synthesis—was downregulated, showing diminished readily available sugar sources for the bioenergetic support of heat-stressed bGCs. Interestingly, four amino acids including L-leucine, lysine, L-tyrosine, and glutamine were upregulated, which points towards further research in the direction of their possible role and mechanisms in cellular protection under stress conditions.

D-Galactosamine (Glan), with structural similarity to hexose sugar, was downregulated in heat stress. Glan is classically involved in liver injury by inducing the apoptosis of hepatocytes [61,62]. Uridine, which is a glycerol-phospholipid and serves as a substrate in phosphatidylcholine (PC) synthesis and is also involved in Pyrimidine metabolism, was downregulated. Interestingly, uridine—as the derivative of Glan—is also involved in the negative regulation of Glan [63]. Glycocyamine, an alpha amino acid related to arginine and creatine metabolism, was downregulated in heat-stressed bGCs. Glycocyamine, as a creatine precursor, has a role in the bioenergetic support of cells in stressful environments [64]. Galactose, an important energy source [65], was down regulated. L-mandelic acid, a monocarboxylic acid involved in the anti-inflammatory process [66], was downregulated. Indole-3-acetaldehyde, an intermediate metabolite in tryptophan metabolism, was also downregulated in heat-stressed bGCs—while the closely related serotonin was also downregulated in heat stress. Indole-3-acetaldehyde is important due to its role in the energy support of stressed cells [67,68]. The metabolite 16-hydroxyhexadecanoic acid, a long chain fatty acid, was downregulated. Xanthine and uridine, being the products of purine degradation [69,70], were downregulated in heat-stressed bGCs—indicating the upregulation of biochemical mechanisms involved in the salvage of purines [71]. The differential metabolite summary results confirm a low bioenergetics microenvironment and higher catabolic activity in response to heat stress.

A univariate ROC analysis of differential metabolites indicated that upregulation of citric acid, choline, 3-hydroxy-3-methylglutaric acid, and glutamine primarily involved the bioenergetics support of heat-stressed bGCs. These metabolites may be considered important biomarkers involved in the eventual salvage of acute heat-stressed bGC’s proliferation potential. Studies support their potential role; for instance, choline and 3-hydroxy-3-methylglutaric acid are shown to be involved in the protection of bovine mammary epithelial cells from acute heat stress [3], and in the support of cell energy metabolism in stress [57], respectively. On the other hand, ciliatine, galactosamine, AICAR, and 16-hydroxyhexadecanoic acid were the acute heat stress-mediated downregulated biomarkers, indicative of signs of diminished anabolic and cell homeostatic processes. For instance, take AICAR, which is an activator of the AMP-activated protein kinase (AMPK) pathway—the AMPK pathway is the sensor of metabolic stress [72], and AICAR-mediated upregulation of AMPK promotes granulosa cell function through the reduction of cytokine-mediated inflammatory processes [73]. Similarly, the correlation and network analysis augmented our results and discussion on the diverse involvement of catabolic processes funneled towards cellular energy support.

Metabolic enrichment and pathway analysis indicated the presence of high energy metabolism. The detail of the metabolites involved in each pathway are comprehensively explained in the respective section. Since we have more or less discussed the same metabolites in the upper section, this section will try to expand on this. Additional information was gained in the pathway analysis concerning choline involvement in “Glycine, serine and threonine metabolism” and “Glycerophospholipid metabolism”, which augments our earlier discussion about the unique multifaceted properties of choline in heat-stressed bGC protection and subsequent cell proliferation. These claims are in accordance with prior studies where choline was able to protect heat-stressed cells [74], and granulosa cells were able to recover from acute and chronic heat stress [32]. The second important metabolite we want to discuss here is uraconic acid, involved in the “Pyrimidine and Histidine metabolism” pathway. There is well-established proof of heat stress-mediated DNA damage and apoptosis in granulosa cells [14,30], and uraconic acid has been shown to protect cells from apoptosis, DNA damage and is involved in DNA damage repair [75,76]. Our metabolism-related pathway enrichment results are more or less in accordance with earlier studies on acute heat-stressed muscle tissues [40,77].

Importantly, this study clarifies that acute heat stress obviously causes decreases in progesterone concentration. This observation of a progesterone decrease is interesting, as studies show conflicting reports concerning progesterone—which sometimes increases and sometimes decreases under the influence of heat stress. Now, at least the question of progesterone in acute heat stress [78] is answered—yes, it decreases. As cholesterol is the master precursor of progesterone, while also acting as a precursor for cholic acid (a bile acid), cholic acid was downregulated in the heat stress group. On the other hand, the main rate limiting precursor of cholesterol synthesis, 3-hydroxy-3-methylglutaric acid, was up-regulated in the heat stress group. Cholesterol was not detected in this study, but the up-regulation of 3-Hydroxy-3-methylglutaric acid and the down-regulation of cholic acid warrants further investigations—both cholic acid and 3-hydroxy-3-methylglutaric acid are important differential metabolites detected in this study. A partial clue to this phenomenon can be presented by the studies referenced here, where cholesterol decreased with acute heat stress [79,80], showing a short half-life and rapid degradation into secondary metabolites [81], a protective role in heat-stressed plants [82], and inducing oxidative stress in neurons [83]. Another important pathway with the largest metabolite enrichment set was “Aminoacyl-tRNA biosynthesis”, which has previously been shown to be significantly enriched in acute heat-stressed muscle tissues [43]. In context with our prior notion concerning the upregulation of certain amino acids, this pathway could be considered further, as all enriched metabolites were amino acids.

## 4. Methods

### 4.1. Granulosa Cell Culture and Heat Treatment

Cyclic ovaries were collected from non-pregnant cows at a local abattoir. Immediately, ovaries were placed in physiologically normal saline (supplemented with 1% antibiotics) and transported to the laboratory. They were washed (3 times) with normal saline and phosphate buffer solution (PBS). Follicular fluid was collected from small healthy follicles (3–8 mm) by an 18-gauge needle, pooled together, sieved through a 40 µm filter (Corning Inc., Corning, NY, USA), and then centrifuged at 1500 rpm for 5 min. Supernatant follicular fluid was discarded, and the bGC pellet was washed (centrifuged at 1500 rpm for 5 min) 2 times with warm PBS containing 1% antibiotics (100 µg/mL Streptomycin and 100 U/mL Penicillin). Cells were seeded (2 × 10^6^ per well) in clear bottom 6-well culture plates (Corning Inc., Corning, NY, USA). Culture media consisted of DMEM/F12 medium (Dulbecco’s Modified Eagle Medium/F-12 Hem; Thermo Fisher Scientific, Waltham, MA, USA) containing 10% FBS (Fetal Bovine Serum; Thermo Fisher Scientific, Waltham, MA, USA) and 1% antibiotic, and culture conditions were 38 °C with 5% CO_2_ (Carbon dioxide) in a humidified cell incubator (Thermo Fisher Scientific, Waltham, MA, USA). Cells were cultured in the same culture medium for 24 h; purity and viability were assessed as described earlier [78]. Culture medium (70%) was changed after 24 h; at 36 h after initial culturing, an 80% confluence of cells was obtained. The culture medium was changed, and cells fasted with culture medium containing reduced FBS (2%) for 12 h. At 48 h, cells were cultured in a fresh original culture medium for an additional 6 h and subsequently subjected to 2 h of acute heat stress (43 °C) in a separate incubator, while the control group remained at 38 °C.

### 4.2. Physical Parameters of Acute Heat-Stressed Granulosa Cells

Cells (2 × 10^4^ in each well) were cultured in 96-well plates and their optical density (OD) was measured using a Cell Counting Kit-8 (CCK-8; Dojindo Laboratories, Kamimashiki-gun, Kumamoto, Japan) The absorbance of the cells was measured by a plate reader after in vitro heat stress (43 °C for 2 h).

Cells were cultured in clear bottom 96-well black plates (Corning Inc., Corning, NY, USA) and their fluorescence optical density (OD) was measured using a 6-carboxy-2′, 7′-dichloro-dihydro-fluorescein diacetate kit (DCFDA kit, Abcam, Cambridge, MA, USA). After 8 h of incubation, fluorescence OD was measured on an Infinite M200 PRO (Tecan Deutschland GmbH, Crailsheim, BW, Germany) fluorescence plate reader.

The qualitative apoptosis rate of the cells was assessed by a fluorescence microscope with an Annexin V-FITC kit (Nanjing Jiancheng Bio Inst., Nanjing, China). Slides were prepared and observed under a fluorescence microscope (Axio Imager A2; ZEISS Microscopy, Oberkochen, BW, Germany). Fluorescence microscope pictures at 40× of early apoptotic (green) and late apoptotic (red) cells were recorded.

### 4.3. Statistical Analysis of Physiological Parameters

The data was taken from at least six replicates each for cell proliferation, ROS, and apoptosis measurements, and were analyzed using IBM SPSS (Statistical Package for Social Sciences software version 26.0, Armonk, NY, USA). Analysis of variance was performed, and means were compared using Tukey’s honestly significant difference (HSD) test at a 5% level of significance (α = 0.05). All the data represented in the figures are expressed as mean ± S.E.

### 4.4. Samples Preparation for LC–MS/MS

After HS treatment, bGCs in 6-well culture plates were allowed to recover for 6 h in an incubator. Then, the culture medium was quickly collected. In micro-centrifuge tubes, 20 μL medium was added to 980 μL cold extraction solvent (50% methanol, 30% acetonitrile, and 20% water). To mix well, the micro-centrifuge tubes were shaken in the Thermomixer at a high speed of 1400 rpm at 4 °C for 10 min, followed by centrifugation at 16,100× *g* for 10 min at 4 °C. The supernatants were transferred to sterile test tubes and kept at −80 °C until liquid chromatography-tandem mass spectrometry (LC–MS/MS) analysis.

Next, 300 μL of ice-cold acetonitrile (Merck, Kenilworth, NJ, USA) was added to 100 μL of the sample, with the addition of 20 μL of 100 μg/mL internal standard, and vortexed for 1 min. Metabolite extraction was performed by maintaining the sample at −20 °C for 20 min; afterwards, the mixture was centrifuged at 13,000 rpm for 5 min under 4 °C. The 300 μL of obtained mixture was nitrogen-dried, and the pellet was re-suspended in 100 μL water (purified using a Milli-Q gradient A10 system) and centrifuged at 13,000 rpm for 5 min under 4 °C. The quality controls (QCs) for investigating the method’s precision were prepared by mixing all samples at equivalent volumes; 3 QCs were ran at the beginning, and an additional QC sample was run for each test sample to monitor the repeatability of the measurements.

### 4.5. LC–MS/MS Analysis and Pre-Processing of Peaks

All extracted samples and QCs were run through LC–MS/MS using HSS T3 100 × 2.1 mm 1.8 μm column (Waters) on an Ultimate 3000 (Thermo Fisher Scientific, Waltham, MA, USA), followed by the analysis employing the Q Exactive system (Thermo Fisher Scientific, Waltham, MA, USA). The positive ion mode (POS-mode) aqueous phase contained 0.1% formic acid (solution A) and 100% methanol (solution B), while the negative ion mode (NEG-mode) contained 10 mM ammonium formate (solution A) and 95% methanol (liquid B). The chromatographic gradient flow rate in both modes was 0.3 mL/min for a total of 20 min, and the column temperature was maintained at 35 °C with an injection volume of 2 μL. Mass spectrometry (MS) was performed at a resolution of 70,000 (Full mass)/17,500 (dd-MS2) method, and an electrospray voltage of 3.8 Kv (POS-mode) and 3.2 kV (NEG-mode), with a capillary temperature of 300 °C.

The original data was converted into a common analysis base file (abf) format via the Reifycs Abf Converter (https://www.reifycs.com/AbfConverter/, accessed on 24 August 2021). Peak identification, retention time correction, and peak area integration were performed using MSDIAL software (http://prime.psc.riken.jp/compms/msdial/main.html, accessed on 24 August 2021). Then, the feature numbers of the sample features were screened to obtain the qualitative results of the sample name, MS1, and MS2, as well as the matrix containing the relative quantification of the peak area. After that, the MSBank and KEGG databases were queried for metabolite identification.

### 4.6. Metabolome Analysis

The MetaboAnalyst 5.0 package [79] was employed to carry out a PCA for investigating clustering trends and outliers. OPLS-DA was performed to identify differential metabolites between the control and heat stress groups [37]. PCA and OPLS-DA were separately performed for NEG and POS mode peak intensity input files. Furthermore, metabolites with VIP values greater than 1 were considered powerful group discriminators between groups and were reported as differential metabolites between the control and heat stress groups. A Students’ *t*-test was also performed to mark these pre-determined differential metabolites between the two groups, employing a significance threshold of *p*-value < 0.05. Biomarker identification and performance evaluation were obtained using ROC curves as calculated by MetaboAnalyst 5.0 [79]. Paired sensitivity and false-positive ratios at different classification decision boundaries were calculated. A ROC curve was plotted with a sensitivity value on the y-axis and the corresponding false positive rate on the x-axis. A correlation heat map of the differential metabolites sets was created in MetaboAnalyst 5.0; *p*-values and correlation coefficient tables were downloaded. Additionally, the correlations between each differential and important differential metabolite group were separately determined through the DSPC algorithm in the MetaboAnalyst 5.0 package. The DSPC algorithm is based on the de-sparsified graphical lasso modeling procedure, useful for discovering correlations among metabolites using lower numbers of samples [80]. DSPC constructs a network of degrees and betweenness based on correlation coefficients and *p*-values for every pair of metabolic features in the input metabolite dataset, where “degree” shows the presence of a correlation and “betweenness” shows the significance-based strength and centrality of a correlation. MetaboAnalyst 5.0 modules of pathway and enrichment analysis were used to determine the biological processes of differential metabolite involvement.

## 5. Conclusions

Heat stress affects the ovarian follicular microenvironment by altering the follicular fluid concentrations of sugars, amino acids, fatty acids, enzymes, antioxidants, and growth factors. Heat stress effects on granulosa cells cause alterations in hormone secretions and disturbs the molecular signaling needed for oocyte development. This study provided evidence of transient acute heat stress characterized by high ROS production, increased rate of apoptosis, diminished progesterone concentration, and time-dependent recovery of cell proliferation in bovine granulosa cells under in vitro culture conditions. Interestingly, bGCs exhibited a time-dependent recovery of cell proliferation activity, which opens the door for further investigations. The remarkable decreases in progesterone observed in the LC–MS analysis and the differential regulation of metabolites related to cholesterol homeostasis require further investigations. Based on the metabolome investigation of acute heat-stressed bGCs, this study proposes choline, citric acid, 3-hydroxy-3-methylglutaric acid, and glutamine as being important upregulated metabolites. Meanwhile, galactosamine, AICAR, ciliatine, 16-hydroxyhexadecanoic acid, succinic acid, uridine, xanthine, and uraconic acid, are important downregulated metabolites. These differential metabolites were implicated in various important metabolic pathways directed towards bioenergetic support mechanisms and the physical adaptations of bGCs under acute heat stress. We propose that the dysregulation of different metabolites involved in the TCA cycle, glyoxylate and dicarboxylate metabolism, and purine and pyrimidine metabolism pathways are of particular interest. The upregulation of certain amino acids and the associated involvement of the Aminoacyl-tRNA biosynthesis pathway also warrants further study in this highly conserved pathway. Further studies targeting these metabolites in conjunction with their related pathways could bring up interesting insights.

## Figures and Tables

**Table 1:** List of differential metabolites selected based on a variable importance in the projection (VIP) score of more than 1. VIP scores were determined through Orthogonal partial least squares discrimination analysis (OPLS-DA), separately for negative (NEG) and positive (POS) ion mode peak lists of the LC–MS/MS analysis. Both fold change (FC) and log2 fold change (log2(FC)) values, along with chemical nature and KEGG compound nomenclature, of the metabolites are given.

Metabolites	Mode	VIP	FC	log2(FC)	Chemical Nature	KEGG
D-(+)-Galactosamine	POS	2.105	0.863	−0.213	Amino acid derivative	C02262
Mycophenolic acid	POS	1.921	1.109	0.149	Aromatic acid	C20380
Choline	POS	1.882	2.157	1.109	Quaternary amine	C00114
Glycocyamine	POS	1.714	0.805	−0.314	Amino acid derivative	C00581
3-Hydroxy-3-methylglutaric acid	NEG	1.701	1.134	0.182	Dicarboxylic acid	C03761
Indole-3-acetaldehyde	POS	1.688	0.815	−0.295	Acetaldehyde	C00637
16-Hydroxyhexadecanoic acid	NEG	1.679	0.767	−0.383	Fatty acid	C13949
Ciliatine	NEG	1.570	0.394	−1.345	Phosphonic acid	C03557
Xanthine	NEG	1.520	0.880	−0.185	Organic compound	C00385
Uridine	NEG	1.502	0.891	−0.167	Ribonucleoside	C00299
Serotonin	POS	1.474	0.855	−0.227	Amino acid	C00780
Citric acid	NEG	1.445	2.157	1.109	Tricarboxylic acid	C00158
Indole-3-carboxyaldehyde	POS	1.440	0.834	−0.263	Organic acid	C08493
Galactose	POS	1.437	0.851	−0.231	Monosaccharide	C00029
L-(−)-Mandelic acid	NEG	1.431	0.931	−0.103	Organic acid	C01984
L-Leucine	NEG	1.410	1.039	0.056	Amino acid	C00123
Lysine	POS	1.393	1.245	0.316	Amino acid	C00047
Pyridoxal	POS	1.369	0.398	−1.329	Organic compound	C00250
Succinic acid	NEG	1.332	0.415	−1.268	Organic compound	C00042
L-allo-Threonine	POS	1.323	1.108	0.147	Alpha-Amino acid	C05519
Proline	POS	1.319	0.848	−0.238	Alpha-Amino acid	C00148
beta-Guanidinopropionic acid	POS	1.316	0.864	−0.210	Alpha-Amino acid	C03065
Progesterone	POS	1.300	0.403	−1.312	Amines	C00410
D-(+)-Pantothenic acid	NEG	1.257	0.868	−0.204	Pantothenic acid	C00864
(−)-Riboflavin	POS	1.249	0.738	−0.438	Organic compound	C00255
Sebacic acid	NEG	1.233	0.944	−0.083	Dicarboxylic acid	C08277
AICAR	NEG	1.221	0.811	−0.303	Peptide	C04677
Cholic acid	NEG	1.189	0.794	−0.332	Bile acid	C00695
4-Nitrophenol	NEG	1.154	1.137	0.185	Phenol	C00870
Glutamine	NEG	1.132	1.131	0.178	Amino acid	C00064
L-Tyrosine	NEG	1.125	1.159	0.213	Amino acid	C00082
L-2-Aminoadipic acid	POS	1.120	0.804	−0.314	Alpha-Amino acid	C00956
Xanthosine	NEG	1.094	0.898	−0.156	Organic compound	C01762
Threonine	NEG	1.076	0.898	−0.155	Amino acid	C00188
Pyridoxine	NEG	1.054	1.042	0.059	Organic compound	C00314
Urocanic acid	POS	1.037	0.666	−0.586	Amino acid derivative	C06559
Thiamine	POS	1.003	0.898	−0.155	Organic compound	C00068

AICAR: 5-Aminoimidazole-4-carboxamide-1-beta-D-ribofuranosyl 5’-monophosphate.

**Table 2:** Correlation-based networks of differential metabolites in heat-stressed bGCs based on the Debiased Sparse Partial Correlation (DSPC) algorithm of the MetaboAnalyst 5.0 software package. Values of degree and betweenness among the metabolite network is shown, where “degree” shows the presence of a correlation and “betweenness” shows the significance-based strength and centrality of a correlation in a network analysis.

Metabolites	Degree	Betweenness	KEGG
Glutamine	20	64.81	C00064
4-Nitrophenol	17	28.59	C00870
Urocanic acid	16	19.06	C00785
L-2-Aminoadipic acid	15	14.37	C00956
D-(+)-Pantothenic acid	14	13.15	C00864
D-(+)-Galactosamine	13	14.85	C02262
Uridine	13	14.64	C00299
L-Leucine	12	18.25	C00123
Xanthosine	12	9.89	C01762
Lysine	10	12.94	C00047
Succinic acid	10	7.97	C00042
Indole-3-acetaldehyde	9	2.36	C00637
Progesterone	8	10.59	C00410
16-Hydroxyhexadecanoic acid	8	7.44	C18218
beta-Guanidinopropionic acid	8	7.31	C03065
Xanthine	7	15.23	C00385
Citric acid	7	13.85	C00158
L-allo-Threonine	7	12.1	C05519
Pyridoxine	7	11.91	C00314
Sebacic acid	7	10.68	C08277
(−)-Riboflavin	7	6.85	C00255
Glycocyamine	7	4.26	C00581
Thiamine	6	5.95	C00378
Pyridoxal	6	1.74	C00250
3-Hydroxy-3-methylglutaric acid	6	1.08	C03761
Proline	5	8.89	C00148
Choline	5	8.78	C00114
Threonine	5	8.58	C00188
Ciliatine	5	5.94	C03557
Galactose	4	7.14	C00984
Mycophenolic acid	4	5.81	C20380

## Data Availability

All the pertinent data is presented in the manuscript and associated Supplementary Files. Raw spectral peaks data can be obtained from the corresponding author.

## Supplementary Materials

The following supporting information can be downloaded at: https://www.mdpi.com/article/10.3390/ijms23042146/s1.
