# Supplementary material for: Investigation of Metabolome Underlying the Biological Mechanisms of Acute Heat Stressed Granulosa Cells
Source: Int J Mol Sci. 2022 Feb 15;23(4):2146. doi: 10.3390/ijms23042146 (PMC8879866; doi:10.3390/ijms23042146)
Supplement: Supplementary file 1 [file ijms-23-02146-s001.zip › ijms-1546287-supplementary.pdf]

Investigation of metabolome underlying the biological mechanisms of acute heat stressed granulosa cells

Supplementary materials:

Supplementary Figure S1: Comparison of physical parameters of bovine granulosa cells (bGCs) exposed to heat stress (43℃) versus control (38℃). bGCs proliferation curves are plotted against different recovery time points in hours (h) for control and heat stress group (A), where MC is the medium change and the time points with arrows shows the significant difference among control and heat stress (HS) groups. Fluorescence OD value (measured at 485/535nm wavelength) of bGCs, stained with 2′,7′-dichlorofluorescine diacetate (DCFDA), is shown on the Y-axis, and the temperature treatments are indicated on the X-axis (B). Representative fluorescence microscope pictures of late apoptotic (red) and early apoptotic (green) cells after staining with FITC/PI dye for control (C) and heat stress treatment (D) groups. Means comparison of apoptotic rate (sum of red and green events) of bGCs under control and heat stress (E). Data are represented as mean ± S.E. of at least three independent cultures with further at least three replicates for each culture. Each panel without common letters is significantly different ($P < 0.05$).

Supplementary Figure S2: The total ion current diagrams of all quality control samples from both positive (POS) ion mode (**A**) and negative (NEG) ion mode (**B**) are superimposed. Spectra overlap with the retention time on X-axis and peak signal intensity fluctuations on Y-axis are shown. The MS2 component matrix box for POS (**C**) and NEG (**D**) ion modes (2,056 and 2,597 MS2 spectra in POS and NEG modes, respectively) from the samples are plotted against the distribution of the mass-to-charge ratio and retention time.

Supplementary Table S1: Differential metabolites ROC (Receiver Operator Characteristic) analysis to determine their biomarker prediction values. Results of AUC (area under curve), *P*-values and FC (fold change) are presented.

Supplementary Table S2: Results of enrichment analysis of the differential metabolites observed in the study.

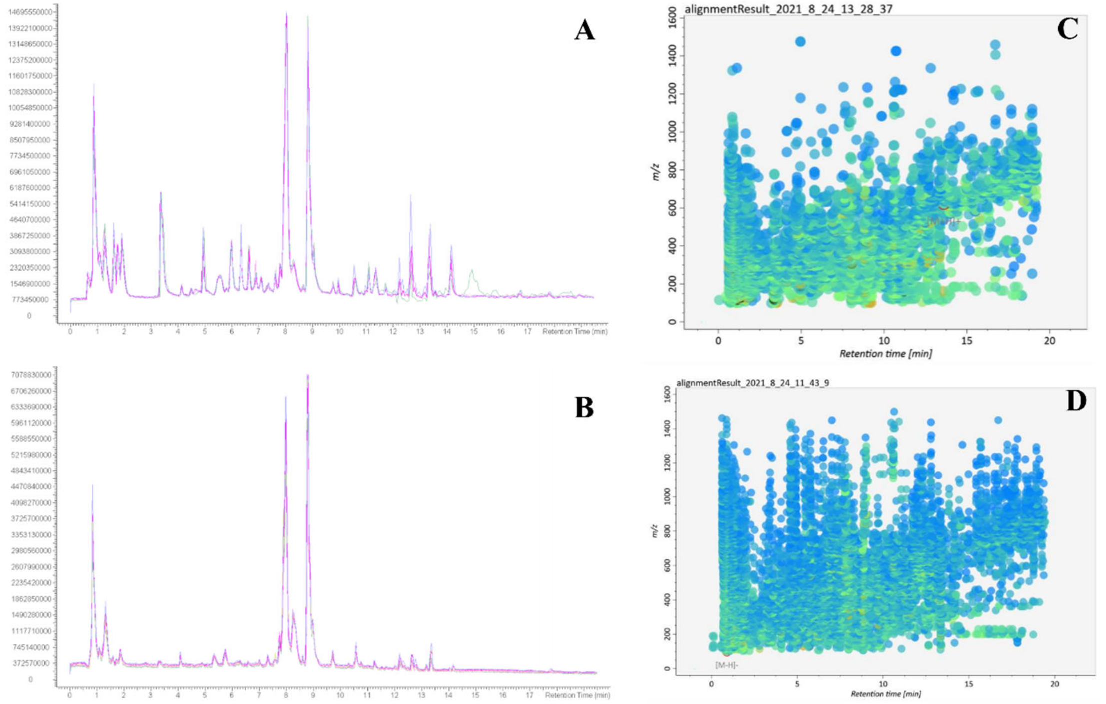
A
B
C
D
Retention Time (min)
alignmentResult_2021_8_24_13_28_37
alignmentResult_2021_8_24_11_43_9
m/z
Retention time [min]
[M-H]-

Figure S1

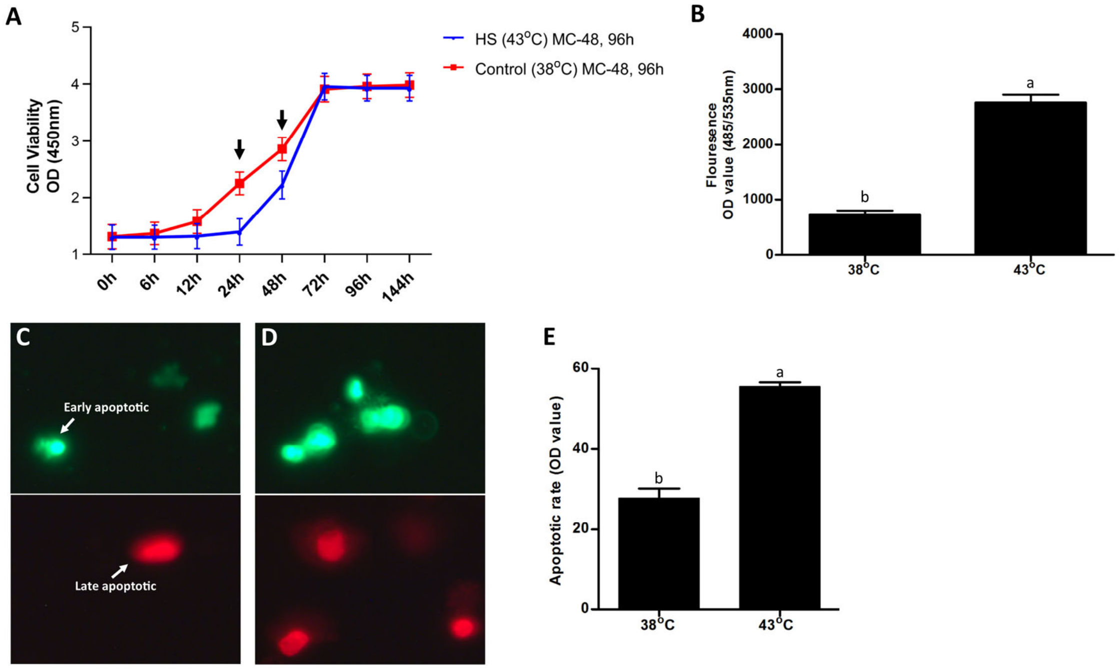

Figure S2

Table S1

Metabolites	AUC	Pval	FC	clusters
Choline	1	0.009	-1.109	5
3-Hydroxy-3-methylglutaric acid	1	0.024	-0.181	5
16-Hydroxyhexadecanoic acid	1	0.0009	0.383	2
D-(+)-Galactosamine	1	0.125	0.212	2
Ciliatine	1	0.014	1.344	2
AICAR	1	0.082	0.302	2
Citric acid	1	0.009	-1.109	5
Glutamine	1	0.032	-0.144	2
Glycocyamine	0.89	0.093	0.313	4
Lysine	0.89	0.154	-0.315	3
Pyridoxal	0.89	0.226	1.3292	4
Proline	0.89	0.288	0.238	4
Mycophenolic acid	0.89	0.278	-0.149	4
4-Nitrophenol	0.89	0.100	-0.185	2
D-(+)-Pantothenic acid	0.89	0.217	0.242	4
Indole-3-acetaldehyde	0.78	0.153	0.295	4
(-)-Riboflavin	0.78	0.188	0.438	4
Sebacic acid	0.78	0.311	0.083	4
L-allo-Threonine	0.78	0.218	-0.147	2
beta-Guanidinopropionic acid	0.78	0.225	0.210	4
Indole-3-carboxyaldehyde	0.78	0.239	0.263	4
Threonine	0.78	0.325	0.155	3

Thiamine	0.78	0.325	0.155	3
Xanthosine	0.78	0.335	0.156	4
L-Tyrosine	0.78	0.199	-0.213	2
Glutamine	0.67	0.471	-0.178	2
L-Leucine	0.67	0.616	-0.056	2
Uridine	0.67	0.920	0.167	2
Galactose	0.67	0.536	-0.170	4
L-(-)-Mandelic acid	0.67	0.727	0.103	4
Progesterone	0.67	0.262	1.312	5
Xanthine	0.67	0.549	0.185	3
L-2-Aminoadipic acid	0.67	0.383	0.314	5
Urocanic acid	0.67	0.399	0.586	5
Succinic acid	0.56	0.544	1.268	3
Cholic acid	0.56	0.513	0.332	1
Pyridoxine	0.56	0.989	-0.059	4

AICAR: 5-Aminoimidazole-4-carboxamide-1-beta-D-ribofuranosyl 5'-monophosphate

Table S2

Differential Metabolites Enrichment Sets			
**Metabolites**	**Total**	**Hits**	**Raw p**
Glycerophospholipid metabolism	36	1	0.0308
Glycine, serine and threonine metabolism	33	3	0.0448
Glyoxylate and dicarboxylate metabolism	32	2	0.0646
Phosphonate and phosphinate metabolism	6	1	0.0855
Ubiquinone and other terpenoid-quinone biosynthesis	9	1	0.1136
Tyrosine metabolism	42	1	0.1136
Phenylalanine metabolism	10	1	0.1136
Phenylalanine, tyrosine and tryptophan biosynthesis	4	1	0.1136
Vitamin B6 metabolism	9	2	0.1754
Biotin metabolism	10	1	0.1973
Steroid hormone biosynthesis	85	1	0.2814
Citrate cycle (TCA cycle)	20	2	0.2876
Aminoacyl-tRNA biosynthesis	48	6	0.2897
Alanine, aspartate and glutamate metabolism	28	3	0.3116
Valine, leucine and isoleucine degradation	40	1	0.3609
Lysine degradation	25	3	0.3639
Arginine biosynthesis	14	1	0.3696
D-Glutamine and D-glutamate metabolism	6	2	0.3696
Nitrogen metabolism	6	1	0.3696
Valine, leucine and isoleucine biosynthesis	8	2	0.4764
Purine metabolism	65	3	0.4794
Histidine metabolism	16	1	0.5130
Pyrimidine metabolism	39	2	0.5520
Thiamine metabolism	7	1	0.6710

Riboflavin metabolism	4	1	0.6964
Primary bile acid biosynthesis	46	1	0.7096
Propanoate metabolism	23	1	0.7351
Butanoate metabolism	15	1	0.7351
Arginine and proline metabolism	38	2	0.8849
Tryptophan metabolism	41	1	0.9744
